# Multiple Structural Maintenance of Chromosome Complexes at Transcriptional Regulatory Elements

**DOI:** 10.1016/j.stemcr.2013.09.002

**Published:** 2013-10-24

**Authors:** Jill M. Dowen, Steve Bilodeau, David A. Orlando, Michael R. Hübner, Brian J. Abraham, David L. Spector, Richard A. Young

**Affiliations:** 1 Whitehead Institute for Biomedical Research, 9 Cambridge Center, Cambridge, MA 02142, USA; 2 Cold Spring Harbor Laboratory, One Bungtown Road, Cold Spring Harbor, NY 11724, USA; 3 Department of Biology, Massachusetts Institute of Technology, Cambridge, MA 02139, USA

## Abstract

Transcription factors control cell-specific gene expression programs by binding regulatory elements and recruiting cofactors and the transcription apparatus to the initiation sites of active genes. One of these cofactors is cohesin, a structural maintenance of chromosomes (SMC) complex that is necessary for proper gene expression. We report that a second SMC complex, condensin II, is also present at transcriptional regulatory elements of active genes during interphase and is necessary for normal gene activity. Both cohesin and condensin II are associated with genes in euchromatin and not heterochromatin. The two SMC complexes and the SMC loading factor NIPBL are particularly enriched at super-enhancers, and the genes associated with these regulatory elements are especially sensitive to reduced levels of these complexes. Thus, in addition to their well-established functions in chromosome maintenance during mitosis, both cohesin and condensin II make important contributions to the functions of the key transcriptional regulatory elements during interphase.

## Highlights

• Cohesin and condensin II occupancy of enhancers and promoters is NIPBL dependent
• Condensin II is recruited to regulatory elements during transcription activation
• Cohesin and condensin II occupy super-enhancers and mediate proper gene expression

## Teaser

The SMC complexes cohesin and condensin II occupy enhancers and promoters in a NIPBL-dependent manner. Condensin II is recruited to active regulatory elements during transcription activation in interphase cells. Both cohesin and condensin II occupy super-enhancers and are required for proper gene expression.

## Introduction

Transcription factors bind regulatory elements such as enhancers and core promoters and interact with cofactors that enable proper control of gene expression (Adelman and Lis, 2012; Lee and Young, 2013; Lelli et al., 2012; Roeder, 2005; Spitz and Furlong, 2012; Zhou et al., 2012). Mediator is an essential coactivator that is recruited to the regulatory regions of most active genes in embryonic stem cells (ESCs) and many other cell types (Kagey et al., 2010). Mediator is bound by NIPBL, which loads cohesin at enhancers and promoters, where this SMC complex contributes to the control of both chromosome structure and gene expression (Dorsett, 2011; Hadjur et al., 2009; Parelho et al., 2008; Phillips and Corces, 2009; Schmidt et al., 2010; Seitan and Merkenschlager, 2012; Wendt et al., 2008). ESCs are highly sensitive to reduced levels of Mediator, NIPBL, cohesin, and another structural maintenance of chromosomes (SMC) complex called condensin II (Fazzio and Panning, 2010; Hu et al., 2009; Kagey et al., 2010). Condensin II is found in the nucleus during interphase where it contributes to interphase chromatin organization (Fazzio and Panning, 2010; Hirota et al., 2004; Ono et al., 2003, 2004) and has been implicated in transcription (Fazzio and Panning, 2010), but its role in gene control is not yet understood. This study reveals that NIPBL-dependent binding of condensin II to promoters and super-enhancers is an integral part of transcription activation in ES cells.

## Results and Discussion

**Figure 1:**
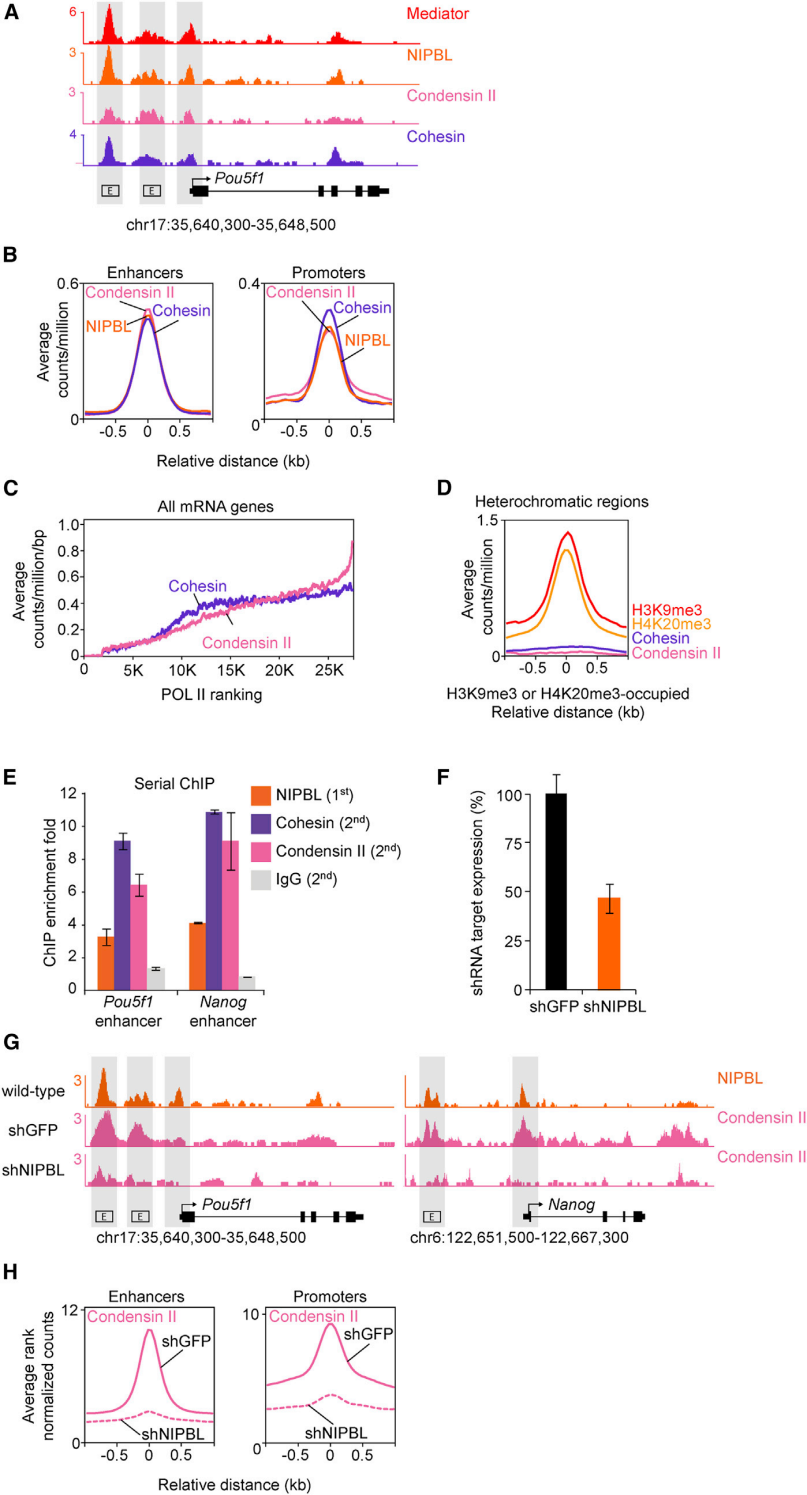
Condensin II and Cohesin Occupy Enhancers and Promoters (A) Binding profiles for Mediator (MED1), NIPBL, condensin II (CAPH2), and cohesin (SMC1) at the *Oct4 (Pou5f1)* locus. ChIP-seq data are shown in reads per million. Characterized enhancer regions (box labeled E) are indicated (Okumura-Nakanishi et al., 2005; Wu et al., 2006; Yeom et al., 1996). (B) Genome-wide distribution of NIPBL, condensin II (CAPH2), and cohesin (SMC1) at enhancers and promoters. Left: enhancer regions defined as regions occupied by OCT4, SOX2, and NANOG (Whyte et al., 2013) are enriched for condensin II, cohesin, and NIPBL. Right: promoter regions defined as regions occupied by TBP and POL II are enriched for condensin II, cohesin, and NIPBL. Metagene representations are centered on the occupied regions, and ±1 kb is displayed. (C) Condensin II and cohesin are associated with mRNA genes with high POL II density. mRNA genes are ranked based on POL II density ±1 kb, and the transcription start site (TSS) and the average number of counts per million per base pair are displayed. (D) Very little condensin II or cohesin signal is observed in regions of the genome containing the heterochromatin marks H3K9me3 or H4K20me3. (E) Serial ChIP showing the presence of NIPBL with condensin II and cohesin. NIPBL was first immunoprecipitated, followed by a peptide elution and a second ChIP for SMC1, CAPH2, or immunoglobulin G. The fold enrichment was determined using RT-QPCR at the *Pou5f1* and *Nanog* enhancer regions. Error bars represent the SD of the average of one to three independent PCRs. p values (at *Pou5f1* enhancer: NIPBL = 0.081, Cohesin = 0.017, and Condensin II = 0.041. At *Nanog* enhancer: NIPBL = 2.9e-3, Cohesin = 2.2e-3, and Condensin II = 0.066) were calculated using a one-tailed t test (Supplemental Information). (F) NIPBL mRNA levels in ESCs infected with shRNA lentiviral constructs targeting GFP (shGFP) and NIPBL (shNIPBL). Transcript levels were normalized to GAPDH. The error bars represent the SD of the average of six independent PCRs. p value (NIPBL = 2.8 × 10^−7^) was calculated using a one-tailed t test. (G) Binding profiles for NIPBL in wild-type ESCs and CAPH2 in ESCs infected with shGFP and shNIPBL at the *Pou5f1* and *Nanog* loci. NIPBL ChIP-seq data are shown in reads per million. For appropriate normalization, CAPH2 ChIP-seq data were rank normalized (Supplemental Information) and represented in rank normalized counts with the y axis floor set to 0.2. (H) Genome-wide distribution of CAPH2 at enhancers and promoters in shGFP- and shNIPBL-treated ESCs. Left: CAPH2 levels are decreased at enhancer regions (one-tailed t test, p < 10^−300^) defined as regions occupied by OCT4, SOX2, and NANOG (Whyte et al., 2013) upon shNIPBL treatment. Right: CAPH2 levels are decreased at promoter regions (one-tailed t test, p < 10^−300^) defined as regions occupied by TBP and POL II upon shNIPBL treatment. See also Figure S1 and Table S1.

To gain insights into the portion of the genome occupied by condensin II in mouse ESCs, we performed chromatin immunoprecipitation of the condensin II subunit CAPH2 followed by massively parallel DNA sequencing (chromatin immunoprecipitation sequencing [ChIP-seq]) (Figure 1). To ensure that this signal represented condensin II complexes, we confirmed the specificity of this antibody (Figures S1A–S1C available online), verified significant overlap with CAPD3 and another CAPH2 antibody (Figures S1D and S1E), and demonstrated that multiple subunits of the condensin II complex interact by ChIP western blot (Figures 1F and 1G). The CAPH2 results showed that condensin II complexes occupy the enhancer and core promoter regions of the well-studied ESC pluripotency gene *Pou5f1* (Figure 1A) and the global population of active enhancers and promoters (Figure 1B), as previously noted for Mediator, cohesin, and the cohesin loading factor NIPBL (Kagey et al., 2010) (Table S1). The enrichment of condensin II and cohesin at genes correlated with the enrichment of RNA Polymerase II (Figure 1C). There were very low levels of condensin II associated with heterochromatin regions (defined by histone H3K9me3 and H4K20me3) (Figure 1D) or insulators (defined by CTCF) (Figure S1H). These results indicate that condensin II occupies transcriptionally active enhancer/promoter regions in ESCs.

The yeast homolog of NIPBL, the Scc2/Scc4 complex, promotes deposition of cohesin and condensin onto chromosomes (Ciosk et al., 2000; D’Ambrosio et al., 2008). Mammalian NIPBL has been shown to recruit cohesin to chromosomes, but it is not known if NIPBL also recruits condensin (Kagey et al., 2010; Watrin et al., 2006). When NIPBL-occupied chromatin fragments were enriched by ChIP, antibodies against both condensin II and cohesin subunits further enriched the enhancers of the pluripotency genes *Pou5f1* and *Nanog* in a second ChIP (Figure 1E), suggesting that NIPBL can occupy the same chromatin fragment with both cohesin and condensin II. To investigate whether NIPBL is required for deposition of condensin II onto chromosome arms, we performed CAPH2 chromatin immunoprecipitation sequencing (ChIP-seq) in ESCs with reduced NIPBL levels due to small hairpin RNA (shRNA) knockdown (Figure 1F). Inspection of the *Pou5f1* and *Nanog* loci revealed that reduced levels of NIPBL led to a reduction in condensin II signal at enhancer and promoter regions (Figure 1G). Genome-wide analysis confirmed that enhancer and promoter regions contained less condensin II signal when NIPBL levels were reduced (Figure 1H). Taken together, these results indicate that NIPBL is necessary for optimal deposition of condensin II at active enhancer/promoter sites. It is thus possible that NIPBL loads condensin II directly, or alternatively, that reduced cohesin loading impacts condensin II loading.

**Figure 2:**
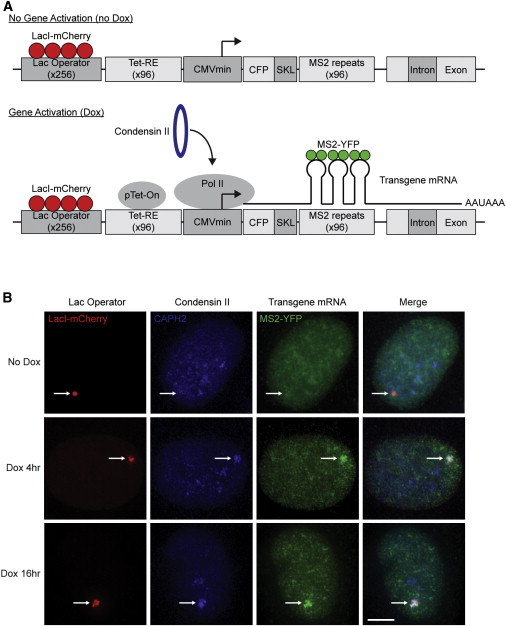
Recruitment of Condensin II at Transcription Activation (A) Schematic representation of the doxycycline (Dox)-inducible transgene integrated at human 1p36 in U20S-2-6-3 cells. LacI-mCherry binds the Lac operator and permits visualization of the locus. Dox is required for pTet-On binding to the Tet response element (Tet-RE). pTet-On binding results in rapid recruitment of POL II and gene activation, as indicated by the MS2-YFP protein. (B) Condensin II is recruited upon transcriptional activation. U20S-2-6-3 cells expressing LacI-mCherry were treated for 4 or 16 hr with vehicle or Dox to induce transcription of the transgene. After crosslinking, cells were labeled with CAPH2 antibody (Ab1). Top: minimal overlap of CAPH2 (blue) with the transgene (LacI-mCherry, red) in the absence of Dox. Middle and bottom: CAPH2 (blue) is recruited to the transgene (LacI-mCherry, red) in the presence of 4 or 16 hr Dox. White arrows point to nuclear localization of the transgene. Representative images (n = 25) are shown. Scale bar, 5 μm.

The presence of condensin II at enhancer/promoter sites occupied by the Mediator coactivator and NIPBL suggests that it is recruited to these sites during transcription activation. To test this model, we used a well-characterized cell system with a stably transfected doxycycline (Dox)-inducible transgene (Figure 2) (Janicki et al., 2004; Zhao et al., 2011). Dox treatment leads to expression of the rtTA (pTet-ON) transcriptional activator, which binds to the transgene locus and rapidly recruits POL II, resulting in expression of the transgene (Janicki et al., 2004; Zhao et al., 2011). Ongoing transcription of the locus can be monitored by the binding of the YFP-tagged MS2 binding protein (MS2-YFP) to MS2 RNA stem loops encoded by the transgene (Janicki et al., 2004; Zhao et al., 2011). The position of the transgene within the nucleus can be visualized by the binding of a fluorescent Lac inhibitor (LacI) protein to a tandem array of lac operator (LacO) repeats at the locus (Figure 2A). The LacI-mCherry signal revealed the location of the transgene in cells with and without Dox treatment (Figure 2B). Condensin II was visualized by immunofluorescence labeling using a specific antibody. In the population of cells examined, there was little colocalization of the condensin II subunit CAPH2 with the inactive transgene (Figure 2B, top row), whereas cells showed a clear CAPH2 signal coincident with the active transgene with 4 (Figure 2B, middle row) or 16 hr (Figure 2B, bottom row) of Dox treatment. These results indicate that condensin II is recruited to newly activated genes in interphase cells.

**Figure 3:**
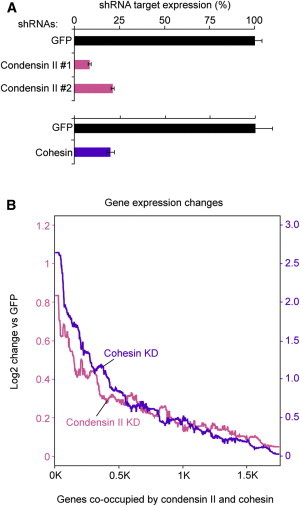
Condensin II and Cohesin Depletions Disrupt the ESC Gene Expression Program (A) CAPH2 and SMC1 mRNA levels in ESCs infected with shRNA lentiviral constructs. Transcript levels were normalized to GAPDH. The error bars represent the SD of the average of three to six independent PCRs. p values (Condensin II #1 = 2e-4, Condensin II #2 = 3e-4, and SMC1 = 1.9e-3) were calculated using a one-tailed t test. (B) Gene expression changes following CAPH2 and SMC1 knockdown at co-occupied genes. Gene expression changes were calculated by comparing the RNA-seq data from cells transduced with condensin II shRNA (left axis) or cohesin shRNA (right axis) to cells transduced with control GFP shRNA. For CAPH2, two highly similar RNA-seq data sets from two different shRNA constructs were pooled. The 1,752 co-occupied genes were ranked based on the average fold change for all shRNAs. See also Figure S2 and Tables S2 and S3.

Loss of ESC identity and viability has been reported following either cohesin or condensin depletion (Fazzio and Panning, 2010; Hu et al., 2009; Kagey et al., 2010). Reduced levels of cohesin cause a disruption in the ESC gene expression program (Kagey et al., 2010), but the effect of reduced levels of condensin II on gene expression remains unclear. We performed RNA-seq transcriptional profiling of ESCs following depletion of condensin II or cohesin (Figure 3; Table S2). We achieved similar knockdown efficiencies for both CAPH2 and SMC1 (Figure 3A; Figure S2A). The expression profiles for cells transduced with two different CAPH2 shRNA constructs were similar to one another (CAPH2 #1 versus CAPH2 #2 Spearman correlation = 0.811) and to those of cells subjected to cohesin depletion (CAPH2 Avg versus SMC1 Spearman correlation = 0.683). Genes cooccupied by condensin II and cohesin were similarly affected by CAPH2 or SMC1 depletion, but the magnitude of change was greater when cohesin was depleted (Figure 3B; Supplemental Information). These observations indicate that reduced levels of condensin II and cohesin lead to a similar disruption of the ESC-specific gene expression program. Although reduced levels of condensin II and cohesin have similar effects on ESC gene expression and identity, other cell types may not have the same requirements for both SMC complexes (Figures 3B and S2B). Cohesin has essential functions in DNA repair, chromosome segregation, and probably gene expression in all cells, whereas condensin II appears to be essential for ESCs but not MEFs (Fazzio and Panning, 2010; Nasmyth and Haering, 2009). It is therefore possible that the requirements for cohesin and condensin II differ in different cells.

**Figure 4:**
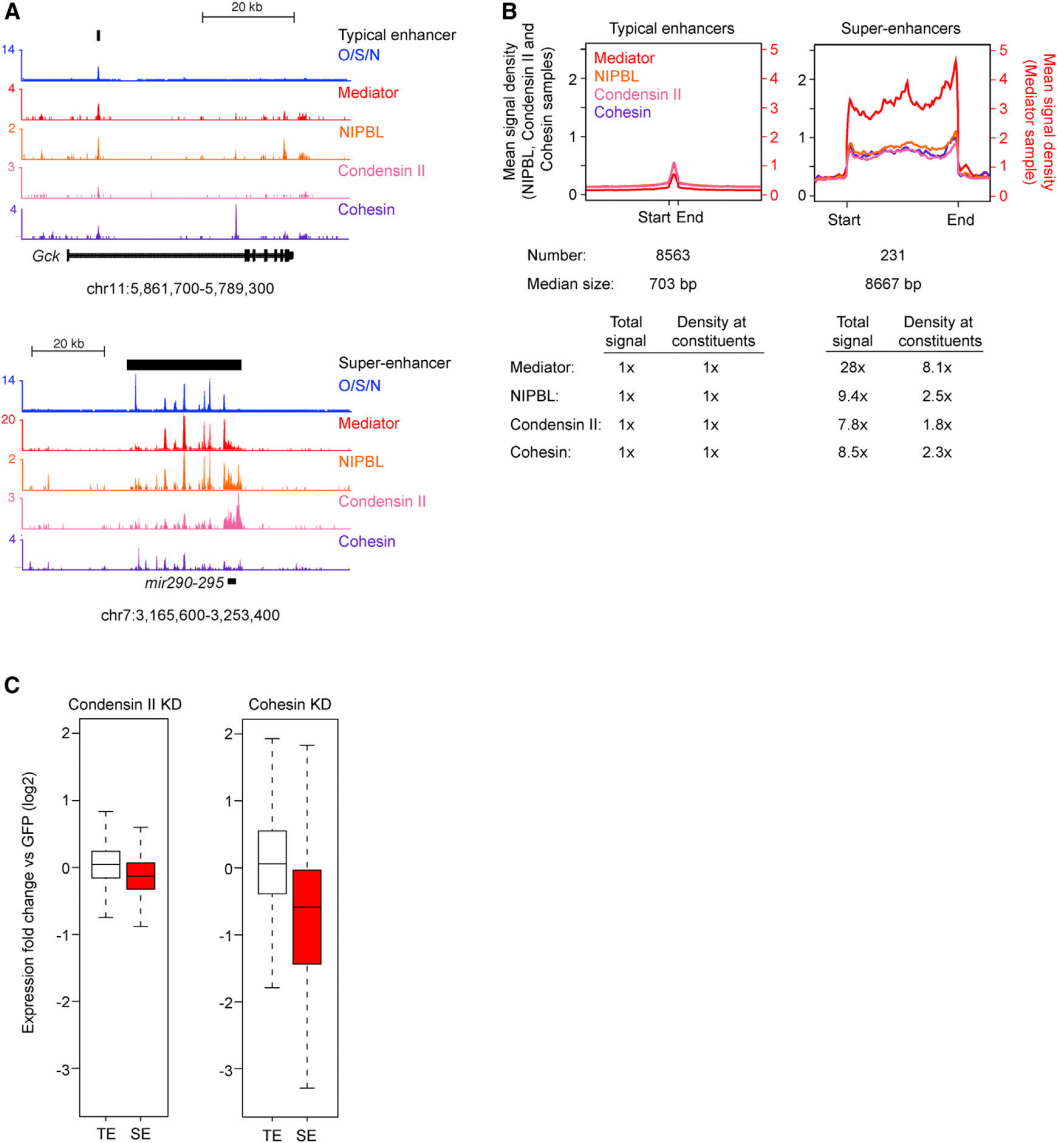
Cohesin and Condensin II Occupy Super-Enhancers and Are Required for Proper Expression of the Key Stem Cell Identity Genes (A) ChIP-seq binding profiles (reads per million) for the ESC transcription factors OCT4, SOX2, and NANOG (OSN), the Mediator coactivator (MED1), NIPBL, condensin II (CAPH2), and cohesin (SMC1) at the *Gck* and *miR-290-295* loci in ESCs. Enhancer bars and scale bars are depicted above the binding profiles. (B) Metagene representations of Mediator, NIPBL, condensin II, and cohesin ChIP-seq density (reads per million per base pair) across the 8,563 typical enhancers and the 231 super-enhancers. Metagenes are centered on the enhancer region (703 base pairs for typical enhancers and 8.7 kb for super-enhancers), with 3 kb surrounding each enhancer region. ChIP-seq fold difference for Mediator, NIPBL, condensin II, and cohesin at super-enhancers versus typical enhancers are displayed below the metagenes. Fold difference at enhancers refers to the mean ChIP-seq signal (total reads) at super-enhancers divided by the mean ChIP-seq signal at typical enhancers. Fold difference at enhancer constituents refers to the mean ChIP-seq density (reads per million per base pair) at super-enhancer constituents divided by the mean ChIP-seq density at typical enhancer constituents. (C) Depletion of condensin II and cohesin caused a decrease in expression of super-enhancer associated genes. Box plots of fold change expression in condensin II and cohesin knockdown cells relative to GFP. Box plot whiskers extend to 1.5× the interquartile range. p values (Condensin II = 7.6e-13 and Cohesin = 2.2e-20) were calculated using a two-tailed t test.

A recently described class of regulatory elements, called super-enhancers, control the expression of key ESC identity genes (Whyte et al., 2013). Super-enhancers consist of clusters of enhancers that are occupied by high levels of Mediator and differ from typical enhancers in size, transcription factor density and content, and sensitivity to perturbation (Whyte et al., 2013). We found that NIPBL and the two SMC complexes are enriched at super-enhancers (Figures 4A and 4B). Like Mediator, there is an increased density of cohesin, condensin II, and NIPBL signal at the 231 ESC super-enhancers compared to the 8,563 typical enhancers (Figures 4A and 4B). Expression of genes with super-enhancers was more sensitive to loss of cohesin and condensin II than genes with typical enhancers (Figure 4C), as observed previously with perturbation of other enhancer-associated factors (Whyte et al., 2013). These results suggest that the loss of ESC gene expression observed with cohesin and condensin II perturbation might be due to direct effects on all affected genes or alternatively, to direct effects at key super-enhancer controlled genes such as those for the pluripotency transcription factors, with consequent secondary effects on most other active ESC genes.

We have shown that both cohesin and condensin II occupy active enhancers and promoters in a NIPBL-dependent manner, that condensin II is recruited to active promoters during transcription activation in living cells, that condensin II is required for normal levels of gene expression, and that both SMC complexes are enriched and function at super-enhancers. Our finding that mammalian condensin II occupies active enhancers and promoters in a NIPBL-dependent manner is consistent with previous studies in yeast showing that both cohesin and condensin require the loading factor for full association with chromosomes (Ciosk et al., 2000; D’Ambrosio et al., 2008). Our observation that mammalian condensin II is recruited to active genes is consistent with work in *Drosophila* where SMC complexes correlate with levels of Polymerase and influence gene expression by facilitating DNA looping (Lupo et al., 2001; Misulovin et al., 2008; Schaaf et al., 2013). Finally, it is interesting that a large fraction of both cohesin and condensin II complexes are loaded at super-enhancers and that reduced levels of these complexes preferentially affect expression of the key cell identity genes associated with these large regulatory domains. Future studies should provide additional insights into the roles of the two different SMC complexes in coordinating transcriptional control with chromosome organization and maintenance.

## Experimental Procedures

A detailed description of all materials and methods can be found in the Supplemental Information.

### ES Cell Culture, shRNA, and Drug Treatment

V6.5 (C57BL/6-129) murine ESCs were maintained under typical mouse ESC conditions on irradiated mouse embryonic fibroblasts (iMEFs) as previously described (Kagey et al., 2010). For ChIP-seq analysis, ESCs were grown two passages off iMEFs. For location analysis following treatment, cells were grown two passages off iMEF feeders and treated with formaldehyde crosslinker. For shRNA-mediated knockdowns, viral media was collected 48 hr after cotransfection with packaging plasmids in 293T cells and ESCs were directly infected. Knockdown ESCs were collected 3 or 5 days postinfection.

### ChIP-Seq and Analysis

ChIPs were performed and analyzed as previously described (Bilodeau et al., 2009; Kagey et al., 2010; Marson et al., 2008). For CAPH2, two antibodies (A302-275A [Ab1] and A302-276A [Ab2], Bethyl Laboratories) were used. For ChIP-seq analysis, reads were aligned with Bowtie (Langmead et al., 2009) and analyzed as described in the Supplemental Information.

### Serial ChIP

For serial ChIP, the first immunoprecipitation was done using the same protocol as a regular ChIP-seq experiment. Following the first immunoprecipitation, beads were eluted twice with 100 μl 2 times; sonication buffer containing 1 mg/ml peptide specific to the antibody for 2 × 30 min. The second ChIP was performed as described in the Supplemental Information.

### Immunofluorescence Assays

U2OS-2-6-3 cells were maintained and transfected as previously described (Janicki et al., 2004; Zhao et al., 2011). For immunofluorescence studies, cells were fixed for 5 min with 2% PFA and incubated overnight at 4°C with CAPH2 antibody Ab1 (1:50, 302-275A, Bethyl Laboratories). Cells were then incubated with a DyLight 405 nm conjugated secondary antibody (Jackson ImmunoResearch Laboratories) for 1 hr at room temperature and mounted in glycerol/DABCO. Maximum intensity projections (40 × 0.2 μm z stacks) of deconvolved images are shown.

## Accession Numbers

ChIP-seq and RNA-seq data have been deposited in Gene Expression Omnibus with accession numbers GSE33346, GSE30919, and GSE46316.

## Supplemental Information

Document S1. Supplemental Experimental Procedures and Figures S1 and S2

Table S1. Enriched Regions, Related to Figure 1

Table S2. Related to CAPH2 and SMC1 Expression Data at Bound Genes, Related to Figure 3

Table S3. Related to CAPH2 and SMC1 Expression Data at Not Bound Genes, Related to Figure 3
