# Supplementary material for: Multiple Structural Maintenance of Chromosome Complexes at Transcriptional Regulatory Elements
Source: Stem Cell Reports. 2013 Oct 24;1(5):371–8. doi: 10.1016/j.stemcr.2013.09.002 (PMC3841252; doi:10.1016/j.stemcr.2013.09.002)
Supplement: Document S1. Supplemental Experimental Procedures and Figures S1 and S2 [file mmc1.pdf]

Stem Cell Reports, Volume *1*

Supplemental Information

Multiple Structural Maintenance of Chromosome Complexes at Transcriptional Regulatory Elements

Jill M. Dowen, Steve Bilodeau, David A. Orlando, Michael R. Hübner, Brian J. Abraham, David L. Spector, and Richard A. Young

CONTENTS

Supplementary Figures

Supplementary Tables

Supplementary Experimental Procedures

Cell Culture Conditions
Embryonic stem cells
U2OS 2-6-3 cells
shRNA-mediated knockdown (Figure 1 and 3)

Chromatin Immunoprecipitation (ChIP)
Antibody specificity (Figure S1)
ChIP protocol
Serial ChIP (Figure 1E)
Immunoprecipitation assays (Figure S1)

ChIP-seq Sample Preparation and Analysis *(Figure 1, 4 and S1)*
Sample preparation
Polony generation and sequencing
ChIP-seq data analysis (Figure 1, 4 and S1)
MACS peak calling method
Metagene representations (Figure 1B, 1D, 1H, S1E and S1H)
ChIP-seq signal at mRNA genes (Figure 1C)
Rank normalization of ChIP-seq data (Figure 1G and 1H)
Super-enhancer analysis (Figure 4)
Assignment of super-enhancers to genes (Figure 4)
Public availability of ChIP-seq datasets

Immunofluorescence *(Figure 2)*

RNA-seq Analysis *(Figure 3 and S2)*
shRNA-mediated knockdown
RNA extraction, cDNA, and TaqMan expression analysis
RNA library preparation
RNA-seq analysis
Public availability of RNA-seq datasets

<u>Supplementary References</u>

Supplementary Figures

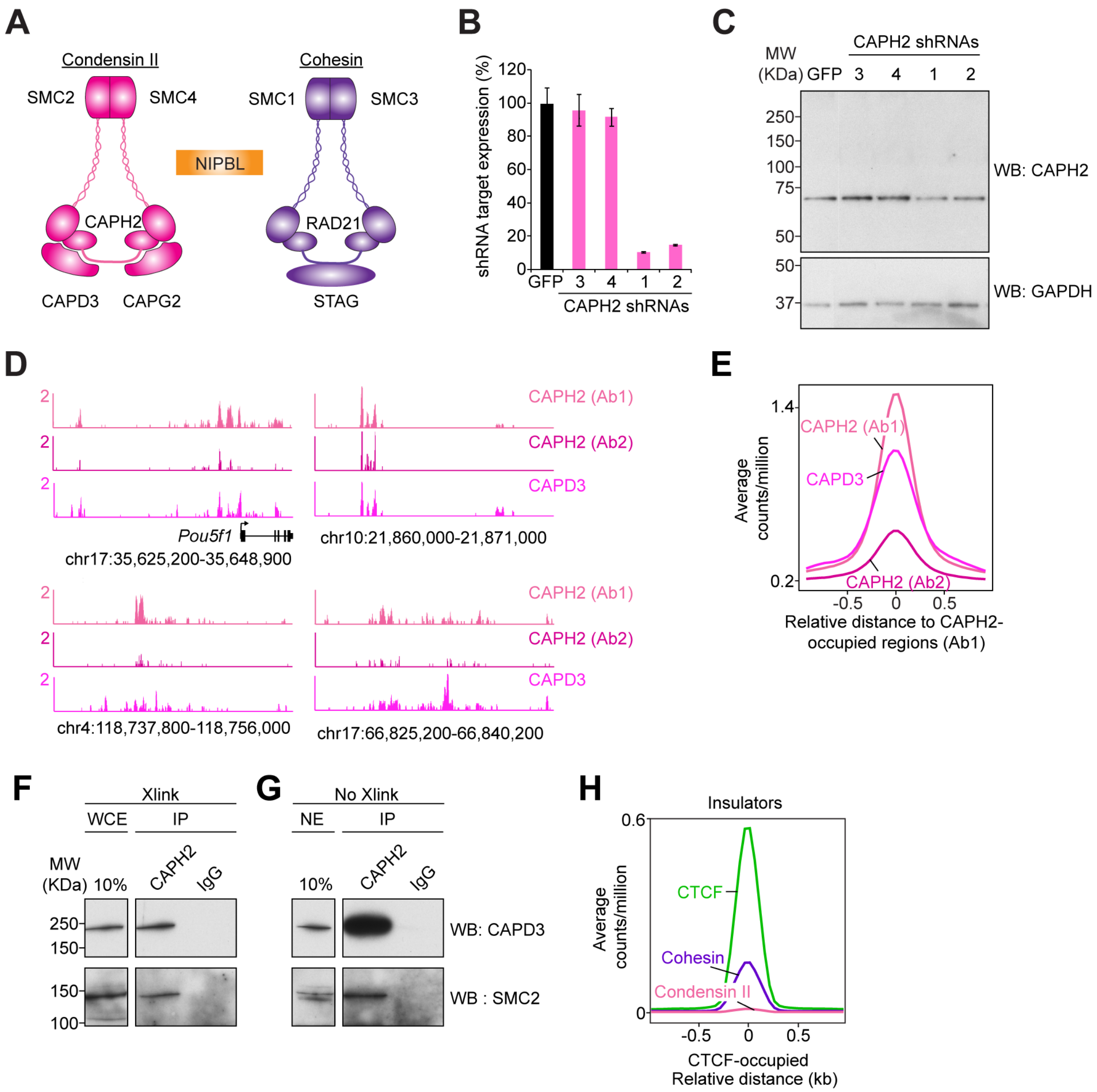

Figure S1: Verification of condensin II signal: validation of the CAPH2 antibody and detection of multiple condensin II subunits. A, Schematic representation of subunits present in condensin II and cohesin complexes. **B,** CAPH2 mRNA levels in ESCs infected with CAPH2 shRNA lentiviral constructs. Transcript levels were normalized to GAPDH. The error bars represent the standard deviation of the average of 2-3 independent PCR reactions. p values (CAPH2 #3 = 0.35, CAPH2 #4 = 0.14, CAPH2 #1 = 1.6e-4 and CAPH2 #2 = 1.8e-4) were calculated using a one-tailed t test. **C,** CAPH2 protein levels in ESCs infected with CAPH2 shRNA lentiviral constructs. The Western blot was performed with the CAPH2 antibody (Ab1). GAPDH (Abcam ab9484) is used as a loading control. **D,** Binding profiles for two condensin II CAPH2 antibodies (CAPH2 Ab1 = Bethyl A302-275A, CAPH2 Ab2 = Bethyl A302-276A) and CAPD3

(Dr. Tatsuya Hirano). Binding profiles are shown for four representative sites in the genome. ChIP-seq data is shown in reads per million with the y-axis floor set to 0.2 reads per million. CAPH2 Ab1 (Bethyl A302-275A) was used throughout this study. **E,** Genome-wide distribution of CAPH2 Ab1, Ab2 and CAPD3 at regions occupied by CAPH2 Ab1. Metagene representations are centered on the occupied regions and ± 1 kb is displayed. **F,** CAPH2 is part of the condensin II complex in ESCs. CAPH2 is precipitated from formaldehyde crosslinked ESC extracts and CAPD3 and SMC2 are detected by Western blot. **G,** CAPH2 interacts with CAPD3 and SMC2. CAPH2 is precipitated from uncrosslinked ESC nuclear extracts and CAPD3 and SMC2 are detected by Western blot. **H,** Genome-wide distribution of CAPH2 and SMC1 at insulator regions defined as sites occupied by CTCF. SMC1 is enriched while CAPH2 is not (55% overlap vs 1%). Metagene representations are centered on the occupied regions and ± 1 kb is displayed.

A

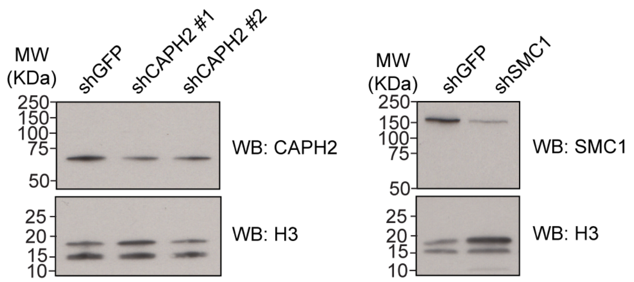

B

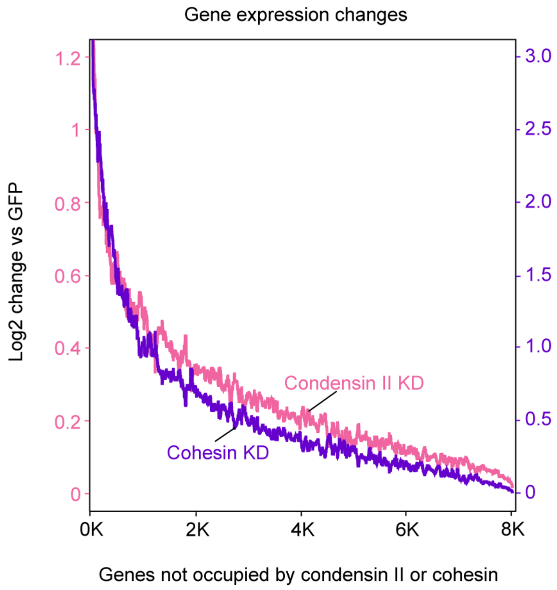

Figure S2: Condensin II and cohesin depletions and gene expression changes at not-occupied genes. A, CAPH2 and SMC1 protein levels in ESCs

infected with CAPH2 shRNA or SMC1 shRNA lentiviral constructs. The Western blots were performed on uncrosslinked nuclear extracts with the SMC1 and CAPH2 (Ab1) antibodies. Histone H3 (Abcam ab1791) is used as a loading control. **B,** Gene expression changes following CAPH2 and SMC1 knockdown of genes not occupied by cohesin or condensin II. Gene expression changes were calculated by comparing the RNA-seq data from cells transduced with condensin II shRNA (left axis) or cohesin shRNA (right axis) to cells transduced with control GFP shRNA. For CAPH2, two highly similar RNA-seq datasets from two different shRNA constructs were pooled. The 8,050 genes not occupied by cohesin or condensin II were ranked based on the average fold change for all shRNAs (Table S3).

Supplementary Tables

Table S1 – Enriched regions
Table S2 – CAPH2 and SMC1 expression data at bound genes
Table S3 – CAPH2 and SMC1 expression data at not bound genes

Supplementary Experimental Procedures

Cell Culture Conditions

Embryonic stem cells
V6.5 murine ESCs were grown on irradiated murine embryonic fibroblasts (iMEFs) unless otherwise stated. Cells were grown under standard mESC conditions as described previously (Kagey et al., 2010). Briefly, cells were grown on 0.2% gelatinized (Sigma, G1890) tissue culture plates in ESC media; DMEM-KO (Invitrogen, 10829-018) supplemented with 15% fetal bovine serum (Hyclone, characterized SH3007103), 1000 U/mL LIF (ESGRO, ESG1106), 100 μM nonessential amino acids (Invitrogen, 11140-050), 2 mM L-glutamine (Invitrogen, 25030-081), 100 U/mL penicillin, 100 μg/mL streptomycin (Invitrogen, 15140-122), and 8 nL/mL of 2-mercaptoethanol (Sigma, M7522).

U2OS 2-6-3 cells
U2OS 2-6-3 cells (Janicki et al., 2004) were grown as described previously in DMEM supplemented with 10% Tetracycline free FBS (Tet System Approved FBS, Clontech), 50 U/ml penicillin and 100 μg/ml streptomycin. Briefly, these cells contain an engineered genetic locus on chromosome 1p36 which can be visualized by the binding of a fluorescent Lac inhibitor (LacI-CFP) protein to a tandem array of lac operator (LacO) repeats. Transcription of the locus can be induced through the Doxycycline inducible reverse tetracycline transactivator (rtTA) binding to Tet operator repeat (Janicki et al., 2004).

shRNA-mediated knockdown (Figure 1 and 3)

Lentiviruses were produced according to Open Biosystems *Trans*-lentiviral shRNA Packaging System (TLP4614). The shRNA constructs are listed below. All are available, including sequences, from Open Biosystems.

CAPH2 #3	TRCN0000173389
CAPH2 #4	TRCN0000174723
CAPH2 #1	TRCN0000175785
CAPH2 #2	TRCN0000175531
GFP	TRCN0000072201
NIPBL	TRCN0000124037
SMC1A	TRCN0000109033

Briefly, mESCs were split off iMEFs, placed in a tissue culture dish for 45 minutes to selectively remove the MEFs and then plated in 6-well plates (300,000 cells/well). The following day cells were infected in ESC media containing 8 µg/ml polybrene (Sigma, H9268-10G). After 24 hours the media was removed and replaced with ESC media containing 4 µg/mL puromycin (Sigma, P8833). ESC media with puromycin was changed daily. Three or five days post infection RNA was extracted and cells were crosslinked for Western blot analysis.

RNA utilized for real-time qPCR was extracted with TRIzol according to the manufacturer protocol (Invitrogen, 15596-026). Purified RNA was reverse transcribed using Superscript III (Invitrogen) with oligo dT primed first-strand synthesis following the manufacturer protocol.

Knockdown efficiency was validated by real-time qPCR carried out on the 7000 ABI Detection System using the following Taqman probes according to the manufacturer protocol (Applied Biosystems).

CAPH2	Mm00518017_g1
SMC1A	Mm01253647_m1
NIPBL	Mm01297461_m1
GAPDH	Mm99999915_g1

Chromatin Immunoprecipitation (ChIP)

Antibody specificity (Figure S1)
For CAPH2-occupied genomic regions, we performed ChIP-seq experiments using Bethyl rabbit polyclonal antibodies A302-275A (Ab1) and A302-276A (Ab2). The antibodies were raised against human CAPH2 (NP_689512.2) residues 425 to 475 (A302-275A) and residue 555 to 605 (A302-276A). CAPH2 Ab1 was used for all ChIP experiments presented in the manuscript. CAPD3 antibody was a generous gift from Dr. Tatsuya Hirano (Ono et al., 2003).

Antibody specificity was confirmed by shRNA-mediated knockdown (Open Biosystems) of CAPH2 mRNA, followed by Western blot analysis. Knockdowns were carried out using the CAPH2 shRNAs according to the manufacturer's protocol (Open Biosystems). Cells were infected with indicated short hairpins for 3 days, followed by RNA extraction, protein extraction and Western blotting.

SMC1 (Kagey et al., 2010), NIPBL (Kagey et al., 2010), POL II (Rahl et al., 2010) and MED1 (Whyte et al., 2013) antibodies have been described previously.

ChIP protocol
Protocols describing chromatin immunoprecipitation materials and methods have been previously described (Bilodeau et al., 2009; Boyer et al., 2005; Boyer et al., 2006; Kagey et al., 2010; Lee et al., 2006; Marson et al., 2008; Rahl et al., 2010). V6.5 mESCs were grown to a final count of 50-100 million cells for each ChIP experiment. Cells were chemically crosslinked by the addition of one-tenth volume of fresh 11% formaldehyde solution for 15 minutes at room temperature. Cells were rinsed twice with 1X PBS and harvested using a silicon scraper and flash frozen in liquid nitrogen. Cells were stored at –80°C prior to use. Cells were resuspended, lysed in lysis buffers and sonicated to solubilize and shear crosslinked DNA. Sonication conditions vary depending on cells, culture conditions, crosslinking and equipment.

For all ChIPs, the sonication buffer was 20mM Tris-HCl pH8, 150mM NaCl, 2mM EDTA, 0.1% SDS, 1% Triton X-100. We used a Misonix Sonicator 3000 and sonicated at approximately 24 watts for 10 x 30 second pulses (60 second pause between pulses). Samples were kept on ice at all times. The resulting whole cell extract was incubated overnight at 4°C with 100 ul of Dynal Protein G magnetic beads that had been pre-incubated with approximately 10 ug of the appropriate antibody. Beads were washed 1X with sonication buffer, 1X with 20mM Tris-HCl pH8, 500mM NaCl, 2mM EDTA, 0.1% SDS, 1%Triton X-100, 1X with 10mM Tris-HCl pH8, 250mM LiCl, 2mM EDTA, 1% NP40 and 1X with TE containing 50 mM NaCl.

Bound complexes were eluted from the beads (50 mM Tris-HCL, pH 8.0, 10 mM EDTA and 1% SDS) by heating at 65°C for 1 hour with occasional vortexing and crosslinking was reversed by overnight incubation at 65°C. Whole cell extract DNA reserved from the sonication step was also treated for crosslink reversal.

Serial ChIP (Figure 1E)
For serial ChIP, the same conditions as standard ChIP were used. For the first immunoprecipitation, 10 Nibpl ChIPs with the equivalent of 100 million cells each were used. Following washes, beads were eluted twice with 100ul sonication buffer containing 0.1 mg/ml of blocking peptide (Bethyl Laboratories, BP302-275). For the second immunoprecipitation, the eluate was divided into 3 different immunoprecipitations (CAPH2, SMC1 and IgG). Washes and final elution were done following the previously described ChIP protocol. Serial ChIP p-values

reported in Figure 1 were determined by using a one-tailed t-test. For the *Nanog* enhancer, p-values were achieved by randomly generating two IgG values, assuming a mean of the measured 0.87 and a standard deviation equal to the SD observed in the IgG sample from the *Pou5f1* enhancer data. This was repeated 10 times and the average p-value is reported.

Immunoprecipitation assays (Figure S1)
Immunoprecipitation assays were performed as previously described (Kagey et al., 2010) using ESCs treated with and without formaldehyde crosslinker. Briefly, for immunoprecipitation with crosslinker, ESCs were processed as a regular ChIP experiment. Following washes, beads were resuspended in gel loading buffer containing 100mM DTT and boiled for 20 minutes. For co-immunoprecipitation without crosslinker, nuclear extracts of ESCs grown for 2 passages off iMEFs were used. The following antibodies were used for Western blot: CAPD3 (A300-604A, Bethyl laboratories) and SMC2 (A300-057A, Bethyl laboratories).

ChIP-seq Sample Preparation and Analysis *(Figure 1, 4 and S1)*

All protocols for Illumina/Solexa sequence preparation, sequencing and quality control were provided by Illumina. A brief summary of the technique and minor protocol modifications are described below.

Sample preparation
DNA was prepared for sequencing according to a modified version of the Illumina/Solexa Genomic DNA protocol. Fragmented DNA was prepared for ligation of Solexa linkers by repairing the ends and adding a single adenine nucleotide overhang to allow for directional ligation. A 1:100 dilution of the Adaptor Oligo Mix (Illumina) was used in the ligation step. A subsequent PCR step with limited (18) amplification cycles added additional linker sequence to the fragments to prepare them for annealing to the Genome Analyzer flow-cell. After amplification, a narrow range of fragment sizes was selected by separation on a 2% agarose gel and excision of a band between 150-350bp (representing shear fragments between 50 and 250nt in length and ~100bp of primer sequence). The DNA was purified from the agarose and diluted to 10nM for loading on the flow cell.

Polony generation and sequencing
The DNA library (2-4 pM) was applied to the flow-cell (8 samples per flow-cell) using the Cluster Station device from Illumina. The concentration of library applied to the flow-cell was calibrated such that polonies generated in the bridge amplification step originate from single strands of DNA. Multiple rounds of amplification reagents were flowed across the cell in the bridge amplification step to generate polonies of approximately 1,000 strands in 1μm diameter spots. Double stranded polonies were visually checked for density and morphology by

staining with a 1:5000 dilution of SYBR Green I (Invitrogen) and visualizing with a microscope under fluorescent illumination. Validated flow-cells were stored at 4 degrees C until sequencing.

Flow-cells were removed from storage and subjected to linearization and annealing of sequencing primer on the Cluster Station. Primed flow-cells were loaded into the Illumina Genome Analyzer II. After the first base was incorporated in the Sequencing-by-Synthesis reaction, the process was paused for a key quality control checkpoint. A small section of each lane was imaged and the average intensity value for all four bases was compared to minimum thresholds. Flow-cells with low first base intensities were re-primed and if signal was not recovered the flow-cell was aborted. Flow-cells with signal intensities meeting the minimum thresholds were resumed and sequenced for 36 cycles.

ChIP-seq data analysis (Figure 1, 4, and S1)
Images acquired from the Illumina/Solexa sequencer were processed through the bundled Solexa image extraction pipeline which identified polony positions, performed base-calling and generated QC statistics.

ChIP-seq reads were aligned using the software Bowtie (Langmead et al., 2009) to NCBI build 37 (mm9) of the mouse genome with default settings. Sequences uniquely mapping to the genome with zero or one mismatch were used in further analysis. When multiple reads mapped to the same genomic position, a maximum of two reads mapping to the same position were used. ChIP-seq datasets profiling the genomic occupancy of OCT4/SOX2/NANOG (Whyte et al., 2013), MED1 (Whyte et al., 2013), SMC1 (Kagey et al., 2010), CTCF (Chen et al., 2008), RNA Polymerase II (Rahl et al., 2010), TBP (Kagey et al., 2010) and H3K9me3 (Bilodeau et al., 2009) in mouse ESCs were obtained from previous publications. Below is the list of ChIP-seq datasets used and corresponding GEO Accession numbers.

Dataset	GEO Accession Numbers	Total Reads
CAPH2	GSM766450, GSM766451	26567296, 27026854
CAPD3	GSM824840	31152087
CAPH2 (shGFP)	GSM824836	31381872
CAPH2 (shNIPBL)	GSM824837	33218700
WCE mES	GSM307154, GSM307155, GSM560357	6514677, 5019381, 7353344
SMC1	GSM560341, GSM560342	8661107, 30429490
POL II	GSM515670, GSM515672	3677488, 13210717

OCT4	GSM1082340	50039788
NANOG	GSM1082342	60867764
SOX2	GSM1082341	68073353
NIPBL	GSM560349,<br>GSM560350	24897794,<br>30323244
TBP	GSM555160,<br>GSM555162	26879855,<br>7705568
MED1	GSM560348	25597506
CTCF	GSM288351	16542968
H3K9me3	GSM459276,<br>GSM459277,<br>GSM459278,<br>GSM459279	13170810,<br>4924706,<br>4735954,<br>6832240
H4K20me3	GSM656527	14325746

All ChIP-seq datastsets, including those obtained elsewhere, were analyzed using the methods described below.

MACS peak calling method
Aligned reads from replicates were combined and peaks of binding were identified using the MACS 1.4 algorithm with all default parameters for CAPH2, SMC1 and NIPBL. Peaks of other factors were called with default parameters, except for the p-value which was set to 1e-09. A summary of the bound regions for each factor is provided (Table S1).

Metagene representations (Figure 1B, 1D, 1H, S1E and S1H)
Metagene representations were created by calculating average count density per bin at a specified set of regions or genes. For any given specific set of regions, the density of the factor was determined in 50bp bins across each individual region. Representations are centered on the bound region and the y-axis corresponds to the average of all regions in counts per million. The WCE density (background) was subtracted. The average ranked normalized read density in each bin was calculated to make the metagene representations in Figure 1H.

ChIP-seq signal at mRNA genes (Figure 1C)
Genes were ordered by their POL II signal +/-1kb around the TSS. Then CAPH2 and SMC1 signal +/-1kb of TSS is displayed in average counts per million per bp. Median smoothing was applied using a window size of 251 for display.

Rank normalization of ChIP-seq data (Figure 1G and 1H)
In order to compare the levels of CAPH2 between shGFP and shNIPBL treated cells, a quantile normalization method was used. For each antibody, the genomic bin with the greatest ChIP-seq density was identified in the two datasets. The average of these values was calculated and the highest signal bin in each dataset was assigned this average value. This was repeated for all genomic bins from the greatest signal to the least, assigning each the average

ChIP-seq signal for all bins of that rank across all datasets. Normalized reads were used to create browser tracks and metagene representations found in Figure 1G and 1H.

Super-enhancer analysis (Figure 4)
Super-enhancers for mouse V6.5 embryonic stem cells were created as described in (Whyte et al., 2013). Briefly, we took the intersection of MACS-defined peaks for OCT4, SOX2, and NANOG to define super-enhancer constituents. Constituents were stitched together if they were within 12.5kb of each other to create stitched enhancers. Stitched enhancers were ranked by their signal of MED1 (length * density). We scaled the stitched enhancer signal ranks such that the maximum *x* axis is 1 and the maximum *y* axis is zero. We then found the *x* axis point for which a line with a slope of 1 was tangent to the curve. We define enhancers above this point to be super-enhancers, and enhancers below that point to be typical enhancers. The fraction of total ChIP-seq reads at enhancers that map to super-enhancers is as follows: CAPH2 (19%), SMC1 (20%), NIPBL (20%) and MED1 (39%).

Assignment of super-enhancers to genes (Figure 4)
We assigned enhancers to genes defined in the RefSeq (NCBI37/MM9) (Pruitt et al., 2007) gene annotations. To assign each enhancer to a gene, we calculated the distance from the center of the enhancer to the TSS of each gene. The enhancer was then assigned to the closest gene.

Public availability of ChIP-seq datasets
ChIP-seq data have been submitted to the Gene Expression Omnibus Database (http://www.ncbi.nlm.nih.gov/geo/) with accession numbers GSE33346 and GSE30919.

Immunofluorescence *(Figure 2)*
U2OS 2-6-3 cells (Janicki et al., 2004) stably transfected to express the rtTA protein were transiently transfected with 1µg of a plasmid expressing LacI-mCherry and 4µg MS2-YFP. After 48 hours, doxycycline (1µg/ml, Invitrogen) or vehicle control was added to the media for 4 or 16 hours as previously described (Janicki et al., 2004; Zhao et al., 2011). Cells were washed with PBS and crosslinked using 2% para-formaldehyde/PBS (from powder, Electron Microscopy Sciences, Hatfield, PA) for 5 min at room temperature. Then, cells were permeabilized with PBS/0.5% TritonX-100/1% normal goat serum (NGS) for 5 min at room temperature. Unspecific binding of antibodies was blocked by PBS/1% NGS for 15 min at room temperature. Cells were incubated overnight at 4C with a 1:50 dilution of anti-CAPH2 antibody (Ab1, 302-275A , Bethyl laboratories) in PBS/1% NGS, washed for 15 min in PBS/1% NGS and incubated with a DyLight 405nm-conjugated anti-rabbit antibody (1:500 in PBS/1%NGS, Jackson Immuno Research Laboratories, Inc.) for 1 hour at room temperature and mounted in glycerol/DABCO mounting media. For imaging, we used an

Applied Precision DeltaVision Core microscope (Applied Precision, Issaquah, WA) with a 63x 1.4 NA oil immersion lens. Images were recorded (40x0.2μm z-stacks), deconvolved and a maximum intensity projection was performed (Applied Precision SoftWorx software) LacI-mCherry in red, MS2-YFP in green, anti-CAPH2 in blue, white arrows indicate the inducible genetic locus. Representative images (n=25) are shown, scale bar 5μm.

RNA-seq Analysis *(Figure 3 and S2)*

shRNA-mediated knockdown
Lentiviruses were produced according to Open Biosystems *Trans*-lentiviral shRNA Packaging System (TLP4614). The shRNA constructs targeting CAPH2, SMC1 and GFP are listed below. All are available, including sequences, from Open Biosystems.

CAPH2 #1	TRCN0000175785
CAPH2 #2	TRCN0000175531
SMC1A	TRCN0000109033
GFP	TRCN0000072201

Briefly, mESCs were split off iMEFs, placed in a tissue culture dish for 45 minutes to selectively remove the MEFs and then plated in 6-well plates (300,000 cells/well). The following day cells were infected in ESC media containing 8 μg/ml polybrene (Sigma, H9268-10G). After 24 hours the media was removed and replaced with ESC media containing 4 μg/mL puromycin (Sigma, P8833). ESC media with puromycin was changed daily. Five days post infection RNA was extracted and cells were collected for Western blot analysis.

RNA extraction, cDNA, and TaqMan expression analysis
RNA utilized for real-time qPCR was extracted with TRIzol according to the manufacturer protocol (Invitrogen, 15596-026). Purified RNA was reverse transcribed using Superscript III (Invitrogen) with oligo dT primed first-strand synthesis following the manufacturer protocol.

Knockdown efficiency was validated by real-time qPCR using the 7000 ABI Detection System. The following Taqman probes were used according to the manufacturer protocol (Applied Biosystems).

CAPH2	Mm00518017_g1
SMC1A	Mm01253647_m1
GAPDH	Mm99999915_g1

Expression levels were normalized to GAPDH levels. All comparisons were made relative to GFP-infected cells. Knockdown efficiencies were between 70 and 90% for all hairpins.

RNA library preparation
Total RNA extraction was performed using the miRVana miRNA extraction kit (Ambion, AM1560) and the recovered RNA was eluted in 30µl elution buffer. Cell numbers were counted from proxy plates using C-Chip disposable hemocytometers (Digital Bio). An amount of RNA that corresponds to 6.52×10^5 cells was used for subsequent steps. Total RNA concentrations were measured using the NanoDrop ND-1000. Synthetic RNAs (ERCC ExFold RNA Spike-In kit, Ambion, 4456739) were added to each sample based on cell number. For each sample 1.42ul of a 1:10 dilution of the synthetic RNA Mix 1 was added to each RNA sample. Libraries were prepared using the TruSeq RNA Kit version 2 (Illumina, RS-122-2001) according to manufacturer's instructions.

RNA-seq analysis
RNA-seq reads were aligned using the software Bowtie (Langmead et al., 2009) to NCBI build 37 (mm9) of the mouse genome with the settings: -e 70 -k 1 -m 2 -n 2. The RPKM (reads per kilobase per million) for each RefSeq gene was calculated using RPKM_count.py (v2.3.5) counting only exonic reads (-e option).

We then looked at the expression changes of the genes that were bound by both CAPH2 and SMC1. A gene was considered bound if it had an enriched region for that factor (see Table S1) overlapping the region 5kb upstream of the TSS through the gene-body. To calculate log2-fold change for those genes, the RPKM of replicates were averaged together and the log2 of the average value divided by the RPKM of the GFP control sample was calculated. To avoid looking at expression changes for genes that were not expressed, we removed any gene which had an RPKM below 1.0 in the GFP control sample. For the remaining genes, the log2 expression changes are available in Table S2. We also looked at the expression changes of the genes that were not bound by CAPH2 or SMC1 (Figure S2B). A gene was considered not bound if it had no enriched region for either factor (see Table S1) overlapping the region 5kb upstream of the TSS through the gene-body. For these genes, the log2 expression changes are available in Table S3.

Public availability of RNA-seq datasets
RNA-seq data have been submitted to the Gene Expression Omnibus Database (http://www.ncbi.nlm.nih.gov/geo/) with accession number GSE46316.

Supplementary References

Bilodeau, S., Kagey, M.H., Frampton, G.M., Rahl, P.B., and Young, R.A. (2009). SetDB1 contributes to repression of genes encoding developmental regulators and maintenance of ES cell state. Genes Dev *23*, 2484-2489.

Boyer, L.A., Lee, T.I., Cole, M.F., Johnstone, S.E., Levine, S.S., Zucker, J.P., Guenther, M.G., Kumar, R.M., Murray, H.L., Jenner, R.G.*, et al.* (2005). Core transcriptional regulatory circuitry in human embryonic stem cells. Cell *122*, 947-956.

Boyer, L.A., Plath, K., Zeitlinger, J., Brambrink, T., Medeiros, L.A., Lee, T.I., Levine, S.S., Wernig, M., Tajonar, A., Ray, M.K.*, et al.* (2006). Polycomb complexes repress developmental regulators in murine embryonic stem cells. Nature *441*, 349-353.

Chen, X., Xu, H., Yuan, P., Fang, F., Huss, M., Vega, V.B., Wong, E., Orlov, Y.L., Zhang, W., Jiang, J.*, et al.* (2008). Integration of external signaling pathways with the core transcriptional network in embryonic stem cells. Cell *133*, 1106-1117.

Janicki, S.M., Tsukamoto, T., Salghetti, S.E., Tansey, W.P., Sachidanandam, R., Prasanth, K.V., Ried, T., Shav-Tal, Y., Bertrand, E., Singer, R.H.*, et al.* (2004). From silencing to gene expression: real-time analysis in single cells. Cell *116*, 683-698.

Kagey, M.H., Newman, J.J., Bilodeau, S., Zhan, Y., Orlando, D.A., van Berkum, N.L., Ebmeier, C.C., Goossens, J., Rahl, P.B., Levine, S.S.*, et al.* (2010). Mediator and cohesin connect gene expression and chromatin architecture. Nature *467*, 430-435.

Langmead, B., Trapnell, C., Pop, M., and Salzberg, S.L. (2009). Ultrafast and memory-efficient alignment of short DNA sequences to the human genome. Genome Biol *10*, R25.

Lee, T.I., Johnstone, S.E., and Young, R.A. (2006). Chromatin immunoprecipitation and microarray-based analysis of protein location. Nat Protoc *1*, 729-748.

Marson, A., Levine, S.S., Cole, M.F., Frampton, G.M., Brambrink, T., Johnstone, S., Guenther, M.G., Johnston, W.K., Wernig, M., Newman, J.*, et al.* (2008). Connecting microRNA genes to the core transcriptional regulatory circuitry of embryonic stem cells. Cell *134*, 521-533.

Ono, T., Losada, A., Hirano, M., Myers, M.P., Neuwald, A.F., and Hirano, T. (2003). Differential contributions of condensin I and condensin II to mitotic chromosome architecture in vertebrate cells. Cell *115*, 109-121.

Pruitt, K.D., Tatusova, T., and Maglott, D.R. (2007). NCBI reference sequences (RefSeq): a curated non-redundant sequence database of genomes, transcripts and proteins. Nucleic Acids Res *35*, D61-65.

Rahl, P.B., Lin, C.Y., Seila, A.C., Flynn, R.A., McCuine, S., Burge, C.B., Sharp, P.A., and Young, R.A. (2010). c-Myc regulates transcriptional pause release. Cell *141*, 432-445.

Whyte, W.A., Orlando, D.A., Hnisz, D., Abraham, B.J., Lin, C.Y., Kagey, M.H., Rahl, P.B., Lee, T.I., and Young, R.A. (2013). Master transcription factors and mediator establish super-enhancers at key cell identity genes. Cell *153*, 307-319.

Zhao, R., Nakamura, T., Fu, Y., Lazar, Z., and Spector, D.L. (2011). Gene bookmarking accelerates the kinetics of post-mitotic transcriptional re-activation. Nat Cell Biol *13*, 1295-1304.
